# Differentiation of Cartilage Repair Techniques Using Texture Analysis from T_2_ Maps

**DOI:** 10.1177/19476035211029698

**Published:** 2021-07-16

**Authors:** Vladimir Juras, Pavol Szomolanyi, Veronika Janáčová, Alexandra Kirner, Peter Angele, Siegfried Trattnig

**Affiliations:** 1 High-Field MR Centre, Department of Biomedical Imaging and Image-guided Therapy, Medical University of Vienna, Vienna, Austria; 2 Institute of Measurement Science, Slovak Academy of Sciences, Bratislava, Slovakia; 3 TETEC AG, Reutlingen, Germany; 4 CD laboratory for Clinical Molecular MR imaging, Vienna, Austria; 5 Austrian Cluster for Tissue Regeneration, Vienna, Austria; 6 Institute for Clinical Molecular MRI in the Musculoskeletal System, Karl Landsteiner Society, Vienna, Austria

**Keywords:** cartilage repair, articular cartilage, magnetic resonance imaging, knee

## Abstract

**Objective:**

The aim of this study was to investigate texture features from T_2_ maps as a marker for distinguishing the maturation of repair tissue after 2 different cartilage repair procedures.

**Design:**

Seventy-nine patients, after either microfracture (MFX) or matrix-associated chondrocyte transplantation (MACT), were examined on a 3-T magnetic resonance (MR) scanner with morphological and quantitative (T_2_ mapping) MR sequences 2 years after surgery. Twenty-one texture features from a gray-level co-occurrence matrix (GLCM) were extracted. The texture feature difference between 2 repair types was assessed individually for the femoral condyle and trochlea/anterior condyle using linear regression models. The stability and reproducibility of texture features for focal cartilage were calculated using intra-observer variability and area under curve from receiver operating characteristics.

**Results:**

There was no statistical significance found between MFX and MACT for T_2_ values (*P* = 0.96). There was, however, found a statistical significance between MFX and MACT in femoral condyle in GLCM features autocorrelation (*P* < 0.001), sum of squares (*P* = 0.023), sum average (*P* = 0.005), sum variance (*P* = 0.0048), and sum entropy (*P* = 0.05); and in anterior condyle/trochlea homogeneity (*P* = 0.02) and dissimilarity (*P* < 0.001).

**Conclusion:**

Texture analysis using GLCM provides a useful extension to T_2_ mapping for the characterization of cartilage repair tissue by increasing its sensitivity to tissue structure. Some texture features were able to distinguish between repair tissue after different cartilage repair procedures, as repair tissue texture (and hence, probably collagen organization) 24 months after MACT more closely resembled healthy cartilage than did MFX repair tissue.

## Introduction

To circumvent the invasive biopsy and limitations of clinical evaluation used to monitor patients after cartilage repair surgery, a number of magnetic resonance imaging (MRI) methods have been developed and validated as sensitive approaches with which to assess cartilage repair tissue maturation. MRI methods comprise morphological evaluation, typically using semiquantitative scoring systems, such as Magnetic Resonance Observation of Cartilage Repair Tissue (MOCART)^1-3^ and glycosaminoglycan- and collagen-specific quantitative MRI techniques.^4-7^ T_2_ mapping, in particular, is the most often used technique for cartilage repair tissue assessment as it reflects the collagen fiber network organization in cartilage, which is represented by the formation of different zones, which is a positive sign of successful tissue maturation after cartilage repair surgery.^7-9^ Unlike the modern glycosaminoglycan-specific techniques, such as sodium MRI and glycosaminoglycan chemical exchange saturation transfer (gagCEST), it does not require an ultra-high field MR scanner to be clinically feasible.^6,10^ As T_2_ is also sensitive to the loading applied to the cartilage, it has been successfully used for functional cartilage repair evaluation.^11,12^ In a clinical setup, however, cartilage repair tissue is characterized by a mean T_2_ value that can be misleading in some cases as T_2_ mapping is limited in ability to detect the subtle details of the repair cartilage architecture and composition.^13^

In such instances, texture analysis of T_2_ maps may be of great help, as it quantifies the relationship between individual pixels and some features can be potentially translated to imaging biomarkers. Texture analysis is used for the extracting the quantitative parameters to describe of (not only MRI) image texture. In the past years, it has been applied in MRI as a computer-aided diagnostic tool.^14^ As T_2_ in cartilage is predominantly related to water content and collagen content and orientation, T_2_ map texture provides an important information about collagen matrix status. Despite some technical challenges of the texture analysis of cartilage (including flattening of the cartilage and input parameter selection), the texture analysis using a gray-level co-occurrence matrix (GLCM) was successfully used in studying patients with osteoarthritis.^15-17^ There were several attempts to use texture analysis of cartilage on morphological images, but this is very challenging, as the texture of cartilage (i.e., signal differences) are dependent on sequence parameters and MR scanner-related variables (such as receiver gain),^18^ which are highly variable and thus suffer from low reproducibility.

There are different strategies for the surgical repair of articular cartilage lesions.^19^ The choice of the repair type depends on the location and size of the defect. Larger lesions are typically treated with osteochondral allograft transplantation or autologous chondrocyte transplantation (ACT), while smaller lesions are treated with marrow-stimulating techniques (such as microfracture [MFX]), or osteochondral autograft transfer.^20-23^ Matrix-associated autologous chondrocyte transplantation (MACT) is very attractive as it uses the patient’s own chondrocytes for cartilage regeneration with the cells seeded on a scaffold (matrix) and can be used in larger cartilage defects.^24^ Various types of scaffold as carriers for chondrocyte transplantation have been designed and tested with different clinical outcome quality.^25-27^

As T_2_ values are generated based on the interplay between water molecules and collagen fibers, the absolute values create a texture that reflects the collagen organization. Recently, texture analysis became very popular either as an independent tool or as a part of machine-learning algorithms for advanced image analysis from different imaging modalities.^14,28,29^ Despite the complex shape of articular cartilage, texture analysis using a GLCM was successfully used on cartilage T_2_ maps to reveal early stages of cartilage degeneration in osteoarthritis (OA)^16,17,30^ or serve as a marker for risk factors of OA progression.^31^ Along with T_2_ mapping, the feasibility of texture analysis of cartilage was tested on T_1_ mapping^32^ and morphological imaging,^18^ with inconclusive results.

To the best of our knowledge, no study has previously investigated focal cartilage lesions using texture analysis for monitoring of cartilage repair maturation. To this end, we suggested an evaluation pipeline for focal cartilage texture analysis using GLCM, including region of interest (ROI) pre-adjustment and validation of suitable texture features selection. The method was used in 2 patient collectives after 2 different cartilage repair types to investigate the ability to distinguish between the outcomes of repair tissue after the surgical procedures.

## Materials and Methods

### Study Design and Patient Demographics

The study was approved by the appropriate ethics committees and regulatory authorities separately for each individual participating site. The results presented here are derived from the subgroup of patients who participated in the MRI substudy of a prospective, multicenter, randomized, controlled, open-label (blinded MRI reading), phase III study comparing the efficacy and safety of MACT using NOVOCART 3D plus (TETEC AG, Reutlingen, Germany) versus MFX in patients with cartilage defects of the knee. Patients were allocated randomly to the MACT or MFX group in a 2:1 ratio and were to be followed-up for 5 years after cartilage repair surgery. Generally, males and females 18 to 65 years of age (or minors of at least 14 years of age with a closed epiphyseal growth plate) with a localized articular cartilage defect of the femoral condyle or the trochlea of the knee (defect grade of III or IV according to the International Cartilage Repair Society (ICRS) classification; maximum of 2 defects) were eligible for enrollment. The maximum total defect size was limited to 6 cm^2^; minimum defect size was 2 cm^2^.

The total patient population (*n* = 262) consisted of 189 males (72.1%) and 73 females (27.9%). The mean age of the patients was (average ± standard deviation) 39.9 ± 10.6 years. Most of the study patients (233 patients, 88.9%) had 1 single lesion, while 29 patients (11.1%) had 2 lesions. All reported lesions had an ICRS grade of 3 or 4. Most of the cartilage lesions in either treatment group were of traumatic origin (77.4% of the lesions in the MFX group and 78.3% of lesions in the MACT group). Both the total lesion size (MFX group: 3.6 ± 1.3 cm^2^; MACT group: 3.8 ± 1.4 cm^2^) and the mean larger lesion size (MFX group: 3.5 ± 1.2 cm^2^; MACT group: 3.6 ± 1.2 cm^2^) were similar in both treatment groups.

One hundred and ten patients were involved in the MRI substudy (MACT: 75 patients; MFX: 35 patients) and had MRIs performed 3, 12, 24, and 60 months after cartilage repair surgery. For the GLCM analysis presented here (24 months time point), T_2_ maps were available for a total of 79 patients (MACT: 55 patients; MFX: 24 patients) after excluding lesions in patella.

### MR Examination

The protocol consisted of a morphological part (TSE PD, TSE T2w, SE T1w) and T_2_ mapping using a multi-echo multi-slice sequence. T_2_ maps were acquired with the following parameters: orientation, sagittal; slice thickness, 3 mm; echo times, (12.5; 25; 37.5; 50; 62.5; 75; 87.5; 100) ms; acquisition matrix, 320 × 320; field of view, 16 × 16 mm; in-plane resolution 0.5 × 0.5 mm; and total scan time 10:36 minutes. The sequence parameters of the whole MR examination protocol are listed in detail in **Table 1**. The central reading site collected all images, performed T_2_ mapping using a 2-parametric exponential fitting method,^33^ and conducted cartilage repair segmentation and scoring.

**Table 1.** Parameters of the MRI Protocol Used in the Multicenter Trial.

Parameter/Sequence	T_2_ Mapping	TSE PD	TSE T_2_w	SE T_1_w
Orientation plane	Sagittal	Coronal	Sagittal	Sagittal
Slice thickness (mm)	3	3	2	2
Slice spacing (mm)	3.3	3.3	2.2	2.2
Repetition time (ms)	2,000	3,080	3,310	700
Echo time(s) (ms)	12.5; 25; 37.5; 50; 62.5; 75; 87.5; 100	28	12	12
Averages	1	2	3	1
Flip angle (°)	90, 180	180	180	90
Acquisition matrix	320 × 256	448 × 403	381 × 448	448 × 381
Image matrix	320 × 320	896 × 896	448 × 448	448 × 448
Field of view (cm)	16 × 16	16 × 16	16 × 16	16 × 16
Total acquisition time (minutes:seconds)	10:36	4:46	3:55	3:53

TSE = turbo spin echo; SE = spin echo; T_2_w = T_2_ weighting; T_1_w = T_1_ weighting; PD = proton-density weighted.

### Image and Texture Analysis

**Figure 1.**
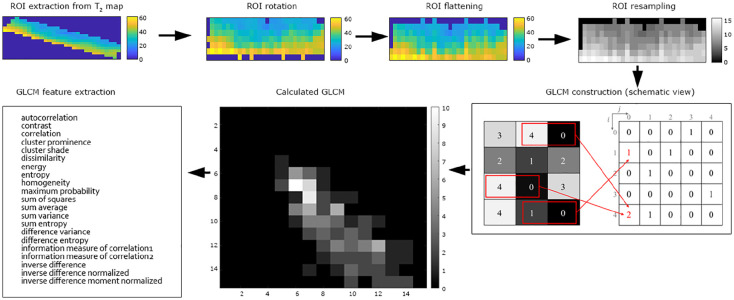
A diagram of texture analysis of cartilage focal lesion.

**Figure 2.**
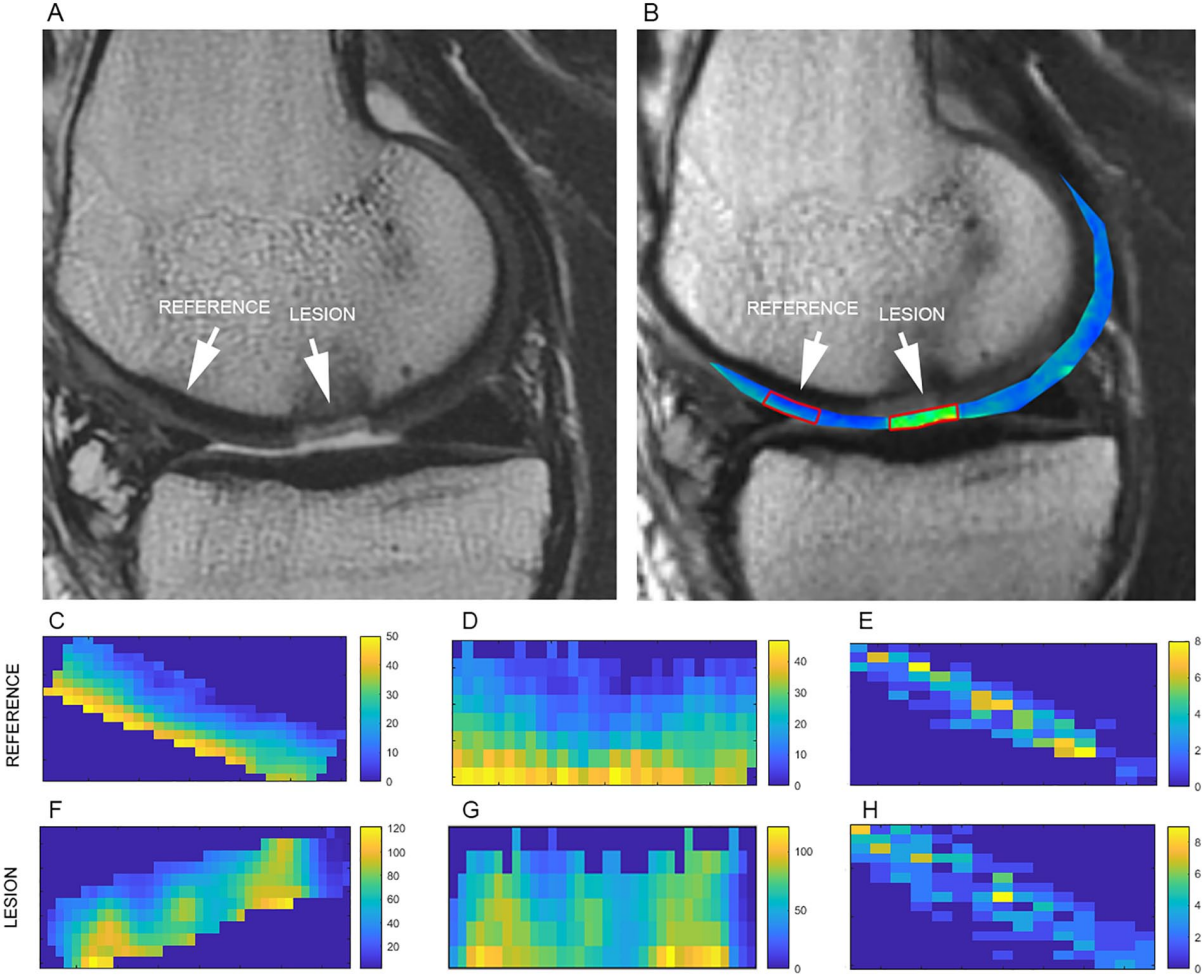
An example of a lesion and reference cartilage ROI selection (**A**—T_2_-weighted turbo-spin echo image; **B**—T_2_ map of segmented cartilage overlaid on the first echo time image); cartilage repair (referred to as the lesion) was selected to include the maximum amount of repaired tissue and a reference cartilage was selected to match the texture of a repair tissue. Extracted ROIs of reference (**C**) and lesion (**F**) were rotated by automatic angle detection (**D**, **G**) and, finally, a GLCM was constructed (**E**, **H**).

Regions of interest were selected on T_2_ weighted images in JiveX (Visus, Bochum, Germany) and subsequently transferred for further processing to MatLab 2020b (Mathworks, Natick, MA). For each patient, 2 to 4 consecutive slices were evaluated to cover the whole cartilage repair site. For each slice, repair cartilage and a reference cartilage were selected by an experienced musculoskeletal radiologist with 28 years of experience (S.T.). As the ROI size was similar between slices, mean T_2_ was calculated and averaged through the slices ROI-wise resulting in 2 T_2_ values per patient (one for the lesion, one for the reference). Zonal T_2_ value of cartilage was calculated as a ratio of superficial and deep zone by dividing the cartilage in halves along the axis perpendicular to cartilage surface. Each ROI was transferred to MatLab and processed with in-house-written scripts. This script automatically loaded the ROIs selected by a reader and performed a texture analysis. The calculation itself took approximately 10 seconds per patient. First, the ROI was automatically rotated (using MatLab function “imrotate” with an argument “orientation” from the function “regionprops”) to be as close as possible to a rectangular shape. Then, GLCM was produced using the *GLCM_features1* function from the MatLab Repository and 21 textural features were extracted (*autocorrelation*, *contrast*, *correlation*, *cluster prominence*, *cluster shade*, *dissimilarity*, *energy*, *entropy*, *homogeneity*, *maximum probability*, *sum of squares*, *sum average*, *sum variance*, *sum entropy*, *difference variance*, *difference entropy*, *information measure of correlation1*, *information measure of correlation2*, *inverse difference*, *inverse difference normalized*, and *inverse difference moment normalized*).^34^ All features were calculated individually for each slice and then averaged. GLCM was processed with the following setup: offset 0° (parallel to the cartilage surface), a direction parallel to the cartilage surface, 16 gray levels, and a step of one pixel. The optimization of GLCM setup (offset, number of gray levels, and step) for focal cartilage texture was done prior to this study and published elsewhere.^35^ The complete ROI processing pipeline prior to texture analysis is depicted in **Figures 1** and **2**.

### Texture Features Reproducibility

To assess the reproducibility of individual texture features, 35 patients were evaluated by 2 independent readers performing GLCM feature selection. To define the impact of ROI selection on the texture features, intra-observer variability was calculated and expressed as a coefficient-of-variation (CV, %) for each parameter, separately for reference and repaired cartilage. In order to quantify the interrelationship between the textural features of cartilage, a cross-correlation matrix was constructed using a paired *t*-test. To evaluate the ability of the texture features to distinguish between reference and repaired cartilage, receiver operation curve (ROC) analysis was used on the entire patient cohort. The area under the curve (AUC) was calculated for each parameter, and interpreted as follows: 0.80 to 1.00 = excellent; 0.60 to 0.80 = good; 0.50 to 0.60 = fair; <0.50 = poor.

### Statistical Evaluation

As a T_2_ map texture of cartilage in weight-bearing and non-weight-bearing zones may differ due to different zonal cartilage stratification, patients were divided into 2 groups according to the repair site location (weight-bearing cartilage = condyle, non-weight-bearing cartilage = anterior condyle/trochlea).

An exploratory analysis of GLCM parameters was performed using a linear model with the fixed categorical effects of “treatment” and “region.” For all parameters, the Shapiro-Wilk test for normality evaluation was used. Treatment differences were estimated using contrast *t*-tests between MACT and MFX for each GLCM parameter. *P* values lower than 0.05 were considered statistically significant.

## Results

**Figure 3.**
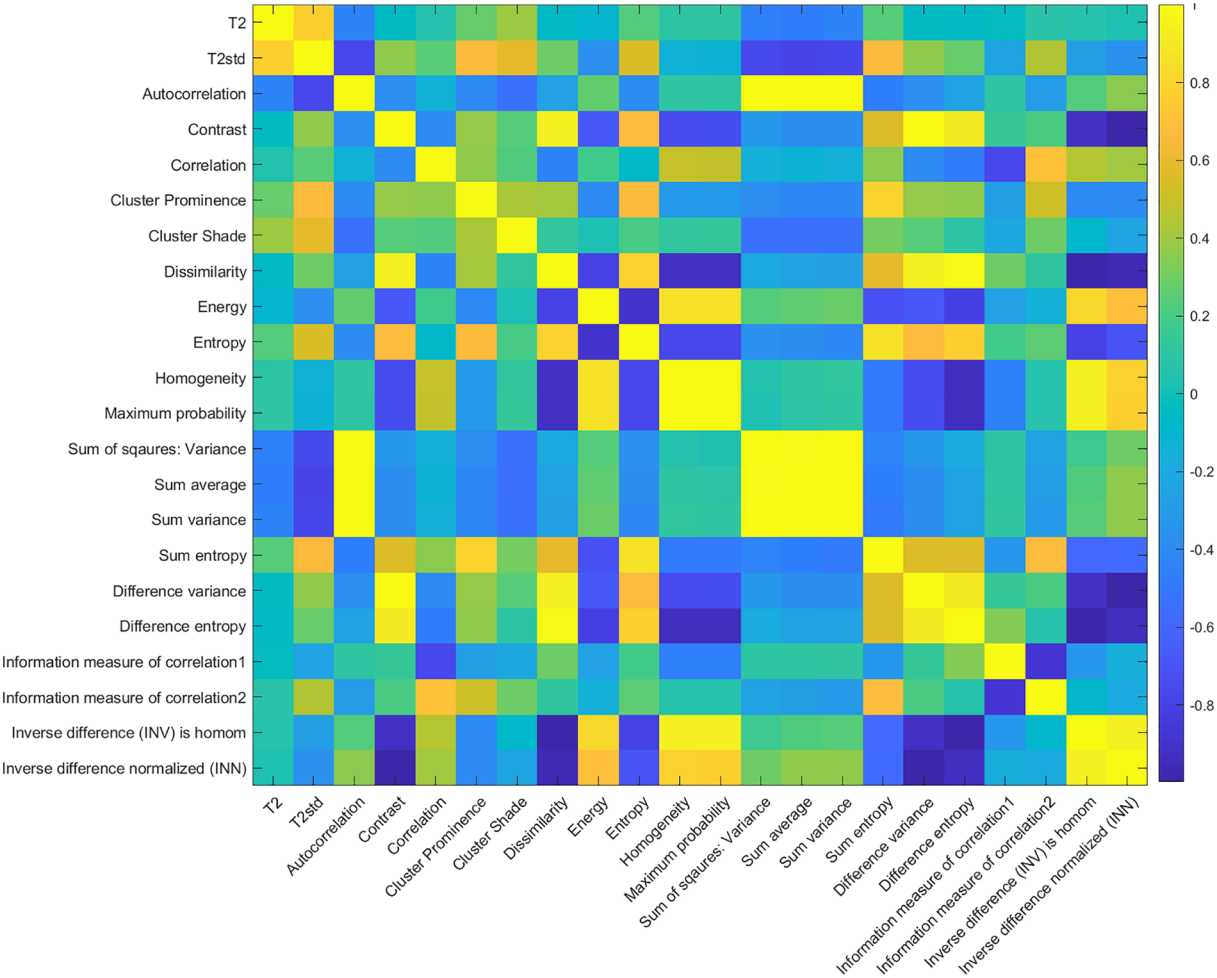
Cross-correlation matrix of T_2_ values, standard deviation of T_2_, and 20 GLCM texture features.

All GLCM features as well as T_2_ values were normally distributed. The interobserver variability showed a low coefficient of variation for T_2_ values and for some texture features (autocorrelation, contrast, correlation, dissimilarity, energy, sum average, sum entropy). For some features, the CV was relatively high (cluster shade [35.79 ± 46.52%] and sum variance [15.15 ± 13.64%]), and in the case of the cluster prominence, even extremely high (516.50 ± 1005.50%). In general, CVs were lower by 1.84% (*P* = 0.344) for the reference cartilage compared to the repair site. ROC analysis revealed moderate to high AUC, and the higher values were recorded in the condyle patient group compared to the anterior condyle/trochlea group. The highest AUC values were found in autocorrelation (0.799), sum of squares (0.810), and sum average (0.795). All CVs and AUC values are listed in **Table 2**. The cross-correlation matrix revealed the interrelationship between autocorrelation and 3 features (sum of squares, sum average, and sum variance). Dissimilarity and contrast were highly correlated as well. The entire cross-correlation matrix is depicted in **Figure 3**.

**Table 2.** Coefficient of Variation (CV [%]) and ROC Calculated for Different Parameters (T_2_ and Texture Features) Extracted from Reference Cartilage and Repair Cartilage.

Parameters	Interobserver Variability				ROC Analysis	
	Repair		Reference		Repair vs. Reference	
	Mean CV	95% CI	Mean CV	95% CI	Condyle (AUC)	Anterior Condyle/Trochlea (AUC)
T_2_	2.31	(2.19, 2.43)	1.78	(1.70, 1.86)	0.540	0.590
Autocorrelation	4.94	(4.74, 5.14)	6.31	(6.09, 6.53)	0.799	0.703
Contrast	0.31	(0.30, 0.32)	0.41	(0.38, 0.44)	0.649	0.660
Correlation	0.02	(0.02, 0.02)	0.02	(0.02, 0.02)	0.529	0.533
Cluster prominence	516.50	(483.91, 549.09)	477.21	(461.12, 493.30)	0.661	0.669
Cluster shade	35.79	(34.72, 36.86)	28.72	(26.90, 30.54)	0.715	0.612
Dissimilarity	0.07	(0.07, 0.07)	0.09	(0.09, 0.09)	0.681	0.676
Energy	0.01	(0.01, 0.01)	0.00	(0.02, 0.02)	0.702	0.689
Entropy	0.09	(0.08, 0.10)	0.09	(0.09, 0.09)	0.633	0.649
Homogeneity	0.02	(0.02, 0.02)	0.02	(0.02, 0.02)	0.703	0.689
Maximum probability	0.01	(0.01, 0.01)	0.01	(0.01, 0.01)	0.695	0.692
Sum of squares	5.11	(4.74, 5.48)	6.29	(5.90, 6.68)	0.810	0.705
Sum average	0.77	(0.75, 0.79)	0.80	(0.74, 0.86)	0.795	0.703
Sum variance	15.15	(14.26, 16.04)	20.18	(19.49, 20.87)	0.799	0.698
Sum entropy	0.04	(0.04, 0.04)	0.05	(0.05, 0.05)	0.714	0.705
Difference variance	0.31	(0.30, 0.32)	0.41	(0.39, 0.43)	0.649	0.660
Information measure	0.02	(0.02, 0.02)	0.02	(0.02, 0.02)	0.630	0.651
Information measure of correlation2	0.01	(0.01, 0.01)	0.01	(0.01, 0.01)	0.533	0.510
Inverse difference normalized INN	0.08	(0.07, 0.09)	0.07	(0.06, 0.08)	0.598	0.608
Inverse difference moment normalized	0.16	(0.17, 0.19)	0.15	(0.14, 0.16)	0.688	0.678

ROC = receiver operation curve; CI = confidence interval; AUC = area under the curve.

From the total number of 57 patients in the condyle group, 16 had MFX treatment, and 41 had MACT treatment. There was no statistical significance between MFX and MACT for T_2_ values (46.57 ± 8.14 ms and 46.22 ± 7.46 ms, respectively, *P* = 0.96) neither for zonal T_2_ analysis (1.11 ± 0.3 a.u. and 1.11 ± 0.5 a.u., respectively, *P* = 0.98). In case of texture features, statistical significance between MFX and MACT was found in autocorrelation (33.71 ± 7.83 and 50.07 ± 5.02, *P* < 0.001), sum of squares (35.63 ± 7.17 and 48.25 ± 4.59, *P* < 0.001), sum average (10.47 ± 1.18 and 12.38 ± 0.76, *P* = 0.01), sum variance (92.00 ± 23.64 and 130.29 ± 15.16, *P* < 0.001), and sum entropy (2.84 ± 0.07 and 2.92 ± 0.04, *P* = 0.05).

From the total number of 22 patients in the trochlea/anterior condyle group, 8 had MFX treatment, and 14 had MACT treatment. There was no statistical significance between MFX and MACT for T_2_ values (46.41 ± 9.5 and 53.25 ± 8.7, respectively, *P* = 0.43) neither for zonal T_2_ analysis (1.12 ± 0.12 a.u. and 1.05 ± 0.09 a.u., respectively, *P* = 0.98). In case of texture features, there was statistical significance between MFX and MACT in dissimilarity (1.52 ± 0.23 and 1.03 ± 0.22, respectively, *P* < 0.001), homogeneity (0.52 ± 0.06 and 0.63 ± 0.06, respectively, *P* = 0.02), and inverse difference normalized INN (0.92 ± 0.02 and 0.94 ± 0.01, respectively *P* = 0.03).

The individual parameters (T_2_ and texture features) are listed for the condyle patient group and the trochlea/anterior condyle patient groups in **Table 3** and **Table 4**, respectively.

**Table 3.** T_2_ and Texture Features in the Condyle Group for Reference and Repair Tissue after MACT and MFX and Statistical Differences between MACT and MFX^a^.

Femoral Condyle	Reference (*N* = 57)		MFX (*N* = 16)		MACT (*N* = 41)		*P* Value (MFX vs. MACT)
	Mean	95% CI	Mean	95% CI	Mean	95% CI	
Global T_2_	44.06	(36.56, 51.56)	46.57	(38.43, 54.71)	46.22	(38.76, 53.68)	0.96
Superficial/deep T_2_	1.13	(1.09, 1.18)	1.11	(0.98, 1.24)	1.11	(0.96, 1.26)	0.98
Autocorrelation	69.94	(64.76, 75.12)	33.71	(25.88, 41.54)	50.07	(45.05, 55.09)	**<0.001**
Contrast	2.84	(2.63, 3.05)	3.15	(2.23, 4.07)	3.44	(2.85, 4.03)	0.57
Correlation	0.88	(0.81, 0.94)	0.80	(0.75, 0.85)	0.86	(0.82, 0.89)	0.05
Cluster prominence	3,138	(2,899, 3,376)	2,702	(2,120, 3,283)	2,927	(2,554, 3,300)	0.49
Cluster shade	14.25	(12.75, 14.94)	107.78	(70.92, 142.81)	72.11	(49.40, 95.49)	0.09
Dissimilarity	1.49	(1.38, 1.60)	1.31	(1.10, 1.52)	1.24	(1.10, 1.38)	0.54
Energy	0.04	(0.03, 0.04)	0.04	(0.04, 0.05)	0.04	(0.03, 0.04)	0.18
Entropy	3.56	(3.30, 3.81)	3.58	(3.46, 3.71)	3.70	(3.62, 3.79)	0.09
Homogeneity	0.66	(0.61, 0.71)	0.55	(0.50, 0.60)	0.59	(0.56, 0.62)	0.12
Maximum probability	0.08	(0.07, 0.08)	0.10	(0.08, 0.12)	0.09	(0.08, 0.10)	0.17
Sum of squares	55.69	(51.64, 59.75)	35.63	(28.46, 42.80)	48.25	(43.66, 52.85)	**<0.001**
Sum average	14.73	(13.59, 15.88)	10.47	(9.29, 11.65)	12.38	(11.62, 13.13)	**0.01**
Sum variance	155.51	(144.18, 166.84)	92.00	(68.36, 115.64)	130.29	(115.13, 145.44)	**<0.001**
Sum entropy	3.03	(2.81, 3.25)	2.84	(2.77, 2.92)	2.92	(2.88, 2.97)	**0.05**
Difference variance	3.89	(3.58, 4.20)	3.15	(2.23, 4.07)	3.44	(2.85, 4.03)	0.57
Difference entropy	1.45	(1.34, 1.56)	1.36	(1.23, 1.49)	1.38	(1.30, 1.46)	0.82
Information measure	−0.39	(−0.36, −0.41)	−0.38	(−0.43, −0.33)	−0.38	(−0.41, −0.35)	0.91
Information measure of correlation	0.90	(0.84, 0.97)	0.90	(0.88, 0.92)	0.90	(0.89, 0.92)	0.68
Inverse difference normalized INN	0.93	(0.92, 0.94)	0.93	(0.92, 0.94)	0.93	(0.92, 0.93)	0.62
Inverse difference moment normalized	0.99	(0.98, 0.99)	0.99	(0.98, 0.99)	0.99	(0.98, 0.99)	0.56

MFX = microfracture; MACT = matrix-associated chondrocyte transplantation; CI = confidence interval.

a Statistically significant differences (*P* < 0.05) are marked in bold.

**Table 4.** T_2_ and Texture Features in the Trochlea/Anterior Condyle Group for Reference and Repair Tissue after MACT and MFX and Statistical Differences between MACT and MFX^a^.

Trochlea/Anterior Condyle	Reference (*N* = 22)		MFX (*N* = 8)		MACT (*N* = 14)		*P* Value (MFX vs. MACT)
	Mean	95% CI	Mean	95% CI	Mean	95% CI	
T_2_	45.21	(41.01, 49.21)	46.41	(36.91, 55.91)	53.25	(44.55, 61.95)	0.43
Superficial/deep T_2_	1.04	(0.94, 1.15)	1.12	(1.00, 1.23)	1.05	(0.96, 1.15)	0.92
Autocorrelation	65.37	(60.62, 70.11)	52.26	(38.96, 65.56)	43.89	(31.66, 56.12)	0.34
Contrast	2.82	(2.61, 3.04)	3.68	(2.62, 4.75)	2.50	(1.53, 3.48)	0.10
Correlation	0.90	(0.83, 0.96)	0.79	(0.72, 0.85)	0.86	(0.80, 0.92)	0.08
Cluster prominence	3,219	(2,969, 3,469)	3,231	(2,376, 4,086)	3,020	(2,233, 3,806)	0.71
Cluster shade	90.60	(72.89, 96.70)	36.34	(−23.11, 94.75)	77.14	(23.06, 131.48)	0.29
Dissimilarity	1.54	(1.43, 1.66)	1.52	(1.29, 1.76)	1.03	(0.81, 1.24)	**<0.001**
Energy	0.06	(0.06, 0.07)	0.04	(0.02, 0.05)	0.05	(0.04, 0.07)	0.10
Entropy	3.29	(3.03, 3.55)	3.71	(3.49, 3.93)	3.47	(3.27, 3.68)	0.12
Homogeneity	0.66	(0.62, 0.71)	0.52	(0.46, 0.59)	0.63	(0.57, 0.69)	**0.02**
Maximum probability	0.10	(0.09, 0.11)	0.08	(0.05, 0.11)	0.11	(0.09, 0.14)	0.14
Sum of squares	42.82	(39.59, 46.06)	50.28	(36.62, 63.94)	44.43	(31.86, 57.00)	0.52
Sum average	15.04	(13.97, 16.11)	12.64	(10.60, 14.68)	11.53	(9.66, 13.41)	0.41
Sum variance	147.12	(135.61, 158.64)	138.86	(97.84, 179.87)	120.61	(82.88, 158.34)	0.50
Sum entropy	2.83	(2.62, 3.03)	2.92	(2.78, 3.06)	2.81	(2.68, 2.94)	0.26
Difference variance	3.91	(3.61, 4.21)	3.68	(2.62, 4.75)	2.50	(1.53, 3.48)	0.10
Difference entropy	1.47	(1.37, 1.58)	1.45	(1.30, 1.60)	1.26	(1.12, 1.40)	0.07
Information measure	−0.50	(−0.46, −0.54)	−0.38	(−0.44, −0.32)	−0.41	(−0.46, −0.36)	0.48
Information measure of correlation	0.90	(0.84, 0.96)	0.90	(0.87, 0.93)	0.90	(0.87, 0.93)	0.93
Inverse difference normalized INN	0.97	(0.95, 0.99)	0.92	(0.90, 0.93)	0.94	(0.93, 0.95)	**0.03**
Inverse difference moment normalized	0.98	(0.98, 0.99)	0.98	(0.98, 0.99)	0.99	(0.99, 0.99)	0.10

MFX = microfracture; MACT = matrix-associated chondrocyte transplantation; CI = confidence interval.

a Statistically significant differences (*P* < 0.05) are marked in bold.

## Discussion

The findings presented in this study showed that texture features based on a GLCM extracted from cartilage T_2_ maps are able to discriminate tissue originating from different cartilage repair methods and potentially can serve as a marker of cartilage repair tissue maturation. Several texture features acquired from GLCM demonstrated high reproducibility, as well as the ability to depict the cartilage texture, especially zonal stratification and overall homogeneity. Unlike T_2_ values, which describe a cartilage tissue with a single mean value over the whole region of interest and, to some extent, a cartilage heterogeneity through standard deviation, texture features reflect the differences of the pixels and their distribution in the ROI.

In this work, some texture features demonstrated relatively high reproducibility in combination with a high AUC, namely, autocorrelation, contrast, homogeneity, sum of squares, sum average, and sum entropy, in particular. This suggests the independence of cartilage lesion and reference selection by differently trained readers, as well as the relatively high ability to distinguish cartilage repair tissue from healthy cartilage. On the other hand, other texture features seem to be extremely dependent on cartilage lesion and reference selection and a small difference in cartilage delineation may result in large discrepancies between readers. It could be due to the fact that it is often challenging to correctly define the cartilage-bone interface on T_2_ maps, as well as the cartilage surface and synovial fluid boundary.

In previously published studies, different selections of texture features were used. To investigate the spatial variation of T_2_ values in cartilage of postmenopausal osteoarthritis patients and age-matched healthy volunteers, Blumenkrantz *et al*. analyzed angular second moment and entropy. Entropy was found to be higher in OA patients, suggesting that T_2_ values in osteoarthritic cartilage are not only elevated but are also more heterogeneous.^15^ Joseph *et al*. investigated patients at risk of OA, and compared to healthy controls, they found higher T_2_ values as well as higher contrast and variance (but not entropy).^31^ Carballido-Gamio *et al*. showed that texture features detected both a different laminar organization and longitudinal laminar changes of cartilage in patients with knee OA. They investigated 2 directions of texture analysis; tendencies showed higher dissimilarity, contrast, energy, and angular second moment perpendicular to the cartilage layers, and higher variance, entropy, homogeneity, and correlation parallel to them.^36^ Texture analysis of T_2_ maps was previously used to predict the need for total knee replacement; contrast was associated with an up to 40% increased risk for total knee replacement in the lateral femur and tibia, and variance in the lateral femur.^37^ Chanchek *et al*. used T_2_ and 3 texture features, variance, contrast, and entropy, to distinguish between diabetes mellitus patients and healthy controls. For all 4 parameters, the statistically significant higher values were found in diabetes mellitus patients, suggesting that this disease negatively influences cartilage tissue.^38^

However, several aspects need to be considered when performing texture analysis of cartilage, such as cartilage-flattening methods, parameter selection from a GLCM (number of gray levels, orientation, and step size), and feature selection and interpretation. As knee articular cartilage is of an extremely irregular shape with various curvatures and thicknesses, it has to be flattened before an actual texture analysis to maintain the selected GLCM direction for all pixels. Flattening methods might have a substantial impact on the resulting texture features. Carballido-Gamio *et al*. compared 3 different flattening approaches: classical reshaping; a parallel method using Bezier spline along the cartilage layers; and a warping method using nonlinear deformation. They found differences in texture features when using different flattening methods; warping was found to be the most appropriate flattening strategy for texture analysis.^16^ An alternative to the flattening methods is the variable angle texture analysis of cartilage using the adaptive offset based on the pixel location within the cartilage.^17^

In our study, the mean T_2_ value was not sensitive enough to distinguish between MFX and MACT repair types (in condyle mean difference T_2_ = +0.32 ms, *P* = 0.96; in trochlea/anterior condyle mean difference T_2_ = +6.84 ms, *P* = 0.43). Zonal T_2_ analysis did not show any significant differences between MFX and MACT either. Previous studies demonstrated the ability of T_2_ mapping to distinguish between cartilage repair in various knee locations,^39^ to monitor the patients during repair maturation^9^ and also to differentiate between repair types.^40,41^ Welsch *et al*. found significantly lower T_2_ values in MFX (47.9 ± 9.8 ms) compared to T_2_ values in MACT (53.6 ± 11.9 ms) and a significantly lower T_2_ index in MFX (0.89 ± 0.12) and MACT (0.99 ± 0.16), and they attributed this difference to more fibrocartilaginous-like characterization of MFX and hyaline-like nature of MACT.^41^ We could not reproduce those findings in our study; one of the reasons could be that they used patients at 36 months after surgery when the tissue differentiation may be more pronounced compared to 24 months follow-up used in our study.

In our study, some of the texture features were significantly different between the 2 repair tissue types (mean differences in condyle, autocorrelation = +16.36 m, *P* < 0.001; sum of squares = +12.62, *P* < 0.001; sum average = +1.91, *P* = 0.01; sum variance = +38.29, *P* < 0.001; and sum entropy = +0.08, *P* = 0.05; in trochlea/anterior condyle, dissimilarity = −0.49, *P* < 0.001; homogeneity = +0.11, *P* = 0.02; and inversion difference normalized INN = +0.02, *P* = 0.03). Comparing these differences to the reference cartilage values, it seems that repair tissue texture (and, hence, probably collagen organization) 24 months after MACT more closely resembles healthy cartilage than does tissue after MFX. However, the interpretation of some texture features with regard to cartilage quality is a difficult task, as some degree of inhomogeneity (zonal stratification) is a characteristic of “normal” cartilage. Autocorrelation seems to be the most reliable feature, as it reflects the repetitive patterns of the texture that can be translated as cartilage zonal stratification. Homogeneity and its inverse counterpart, dissimilarity, are harder to interpret, as it is impossible to determine whether an inhomogeneity results from disorganized collagen fibers or from zonal stratification. Our results suggest, however, that the former inhomogeneity influence is somehow larger, which resulted in the ability of texture-homogeneity to distinguish between repair tissue types.

Our results support earlier findings on inferior repair tissue quality after MFX compared to MACT. In a meta-analysis, MFX was found to produce primarily fibrocartilage.^42^ Hyaline repair tissue was more common with ACT than with MFX.^43^ The inferior quality of repair tissue after MFX, and especially, in larger lesions, as well as a worse defect filling compared to MACT, is considered the reason for long-term inferior clinical results of MFX.^44,45^ In this context, it has been shown that filling of smaller, well-shouldered cartilage defects, even with non-hyaline repair tissue, still improves function and clinical complaints within the first few years, while the histological repair tissue quality becomes more important for larger defects and in the longer term.^43,45-47^ This meta-analysis also found a difference with regard to tissue maturation. While repair tissue from MACT becomes more hyaline-like (a process that takes up to 5 years),^48^ demonstrating tissue maturation with increased stiffness of the repair tissue, the amount of fibrocartilage formed after MFX only enlarges over time, but usually without maturation into cartilage with hyaline properties.

This study had several limitations. First, the sample size was relatively low, particularly the trochlea/anterior condyle group. In theory, these 2 groups could be merged together to increase the statistical sample size, but it would very likely result in lower sensitivity of the calculated texture features, as zonal stratification as well as absolute T_2_ values vary in different cartilage locations. Second, the texture analysis was performed slice-wise rather than in 3D fashion. Three-dimensional texture analysis is definitely more robust compared to 2D,^49,50^ but because of the nonzero slice distance typically used in multi-echo spin-echo T_2_ mapping, it is impossible to implement 3D texture analysis on T_2_ maps.

## Conclusion

In conclusion, texture analysis using GLCM provides a useful add-on to T_2_ mapping for the characterization of cartilage repair tissue by increasing its sensitivity to overall structure. Some texture features, such as autocorrelation, homogeneity, dissimilarity, sum of squares, sum of averages, and sum entropy, were able to distinguish between repair tissue that resulted from MACT and MFX, whereby repair tissue texture (and hence, probably collagen organization) 24 months after MACT more closely resembled healthy cartilage compared with MFX repair tissue. This is in accordance with other publications that have reported better repair tissue quality with ACT compared to MFX. In terms of methodology, it is crucial to individually evaluate weight-bearing and non-weight-bearing cartilage, as their texture substantially differs. The results of this study suggest that using texture analysis in clinical trials monitoring the status of repaired cartilage may provide additional information about the cartilage structure and composition.
